# Prevalence of Vitamin D Deficiency among Adult Population of Isfahan City, Iran

**DOI:** 10.3329/jhpn.v29i2.7857

**Published:** 2011-04

**Authors:** Silva Hovsepian, Massoud Amini, Ashraf Aminorroaya, Peyvand Amini, Bijan Iraj

**Affiliations:** Isfahan Endocrine and Metabolism Research Center, Isfahan University of Medical Sciences, Isfahan, Iran

**Keywords:** Adult, Cross-sectional studies, Parathyroid hormone, Seasonal variation, Vitamin D deficiency, Iran

## Abstract

Determination of vitamin D status in different age-groups in a community and in different climates of a country is necessary and has important implications for general health. The study was conducted to determine the prevalence of vitamin D deficiency among the adult population of Isfahan, a centrally-located city in Iran. In this cross-sectional study, 1,111 healthy people—243 men and 868 women—aged 41.4 (mean 14 and range 20-80) years, who attended a single-consultation outpatient clinic, were selected. Serum 25-hydroxy vitamin D (25-OHD), parathyroid hormone (PTH), calcium and phosphorus concentrations were measured. Mild, moderate and severe vitamin D deficiencies were defined as 25-OHD values of 20-30 ng/mL, 10-20 ng/mL, and <10 ng/mL respectively. The median (range) concentrations of 25-OHD were 21 (4.0-105.0) ng/mL in males and 18 (1.5-117) ng/mL in females (p=0.05). The prevalence of mild, moderate and severe vitamin D deficiencies among the adult population was 19.6%, 23.9%, and 26.9% respectively. Vitamin D deficiency was more prevalent among women (p=0.001) and younger age-group (p=0.001). Medians of 25-OHD in spring-summer and autumn-winter were 21 ng/mL and 18 ng/mL respectively (p=0.005). The prevalence of severe vitamin D deficiency was higher in autumn-winter than in spring-summer (odds ratio=1.6, 95% confidence interval 1.2-2.2, p=0.001). The prevalence of vitamin D deficiency was high in a sunny city—Isfahan— especially among women and younger population. The high prevalence of vitamin D deficiency in this city emphasizes the necessity of vitamin D supplementation as more exposure to sun is limited due to the type of clothing required by current law.

## INTRODUCTION

Vitamin D deficiency is considered to be one of the most common medical conditions worldwide. The consequences of vitamin D deficiency include poor bone development and health and also increased risk of many common and serious diseases, including some common cancers, cardiovascular diseases, type 1 diabetes, and other autoimmune diseases (1, 2). It has been reported that 30-50% of both children and adults in the United States, Canada, Europe, Australia, New Zealand, and Asia are vitamin D-deficient. Despite the important role of sunlight in vitamin D synthesis, recent studies have shown that the rate of vitamin D deficiency is also higher in the sunniest areas of the world, including the Middle East countries, such as Saudi Arabia, Qatar, and United Arab Emirates, Turkey, India, and Iran because of low exposure to sun due to cultural factors (3-13).

Results of studies in Iran among different age-groups indicate a high prevalence of vitamin D deficiency (13-17). Studies in Isfahan have shown a high prevalence of the problem among high school children, pregnant women, and newborns (15-17). Results of a study in Tehran showed that the prevalence of vitamin D deficiency was also higher among the general population (14).

Determination of vitamin D status in different age-groups in a community and in different climates of a country is necessary and has important implications for general health. Since there is already considerable published information on vitamin D deficiency in women, adolescent girls, and children, the present study was conducted to investigate the prevalance of vitamin D deficiency among men and women. We conducted the study in Isfahan, a sunny centrally-located city in Iran.

## MATERIALS AND METHODS

### Study subjects and area

In this cross-sectional study in Isfahan, a sunny city located in the central part of Iran, 1,111 healthy individuals—243 men and 868 women—aged 41.4 (mean 14 and range 20-80) years, who attended a single-consultation outpatient clinic for routine check-up, were consecutively selected. Subjects with known hepatic or renal disease, metabolic bone disease, malabsorption, type 1 diabetes, hypercortisolism, malignancy, immobility for more than one week, pregnancy, lactation, and medications influencing bone metabolism were not eligible for the study.

### Seasons of study period

The samples were taken over a one-year period and were collected at each of two time-points (autumn/winter—October/March and spring/summer—April/September). We have presented both pooled data, and data were analyzed by season.

### Biochemical parameters

Sampling was performed between 8:00 and 9:00 am in the laboratory of the Isfahan Endocrine and Metabolism Research Center. Blood samples were centrifuged and stored at −20 °C. Calcium, phosphorus, 25-hydroxy vitamin D(25-OHD) and parathyroid hormone (PTH) were measured.

Serum 25-OHD was measured by radioimmunoassay (RIA) and PTH by immunoradiometric assay (IRMA) (kits manufactured by Biosource Europe SA, Belgium). Intra-assay and interassay coefficient of variation for 25-OHD was 3.3% and 5.2% respectively, and for PTH these were 4.2% and 6.6% respectively. The normal range for PTH was 10-65 pg/mL.

25-OHD and PTH were measured using autoanalyzer—Liasys (Italy). Serum calcium and phosphorus were analyzed using Pars Azmoon kits (Pars Azmoon Co., Iran) by cresolphthalein complexion method. The normal ranges of calcium and phosphorus were 8.5-10.1 mg/dL and 3.5-5 mg/dL respectively.

### Definition of vitamin D deficiency

Mild, moderate and severe vitamin D deficiencies were defined as 25-OHD values of 20-30 ng/mL, 10-20 ng/mL, and <10 ng/mL respectively.

There is also another classification for vitamin D deficiency in the literature. In this classification, the combination of moderate and severe vitamin D deficiencies is considered vitamin D deficiency (25-OHD <20 ng/mL) and mild vitamin D deficiency (25-OHD 20-30 ng/mL) as vitamin D insufficiency (18). We used the first classification. However, the second classification was used after stating its usage.

### Statistical analysis

Normality of data distribution was assessed with Kolmogrov-Smirnov. Log transformation was used for reducing skewness. Otherwise, the median values were presented for variables which were not normally distributed. For all other variables with normal distribution, data were presented as mean [standard deviation (SD)]. Mean and/or median of the study variables between groups were compared using analysis of variance (ANOVA), Kruskal-Wallis, Wilcoxon test (when appropriate), and Post hoc tests.

Chi-square was used for comparing the frequencies. The differences were considered significant at p values of less than 0.05. Statistical analysis was conducted using the SPSS software (version 15) (SPSS Inc., Chicago, USA).

We determined a cut-off point for mild vitamin D deficiency according to the level of vitamin D and PTH in our population at concentrations of vitamin D from 10 ng/mL to 45 ng/mL, with 5 ng/mL interval, using area under the receiver operating characteristic curve (ROC).

### Ethical issues

All the study subjects gave voluntary informed consent before participation. Those who refused to take part in the study were excluded. The Institutional Review Board and Medical Ethics Committee of the Isfahan University of Medical Sciences approved the protocol.

## RESULTS

During the study, 1,111 healthy residents of Isfahan, aged 20-80 years, were studied. Their mean age was 41.4 (median 14) years (Table). Calcium and phosphate concentrations were normally distributed. However, levels of 25-OHD and PTH were not normally distributed.

**Table.** Mean (standard deviation) and median (range) of biochemical characteristics of adult Isfahani people (n=1,111) in different age-groups

Biochemical variable	20-39 years (n=494)	40-59 years (n=489)	60-80 years (n=128)	p value
Calcium (mg/dL)	9.3 (0.6)	9.3 (0.6)	9.4 (0.7)	NS
Phosphorus (mg/dL)	3.4 (1.0)	3.5 (0.9)	3.5 (1.0)	NS
25-OHD (ng/mL)	14 (2-182)	22 (1.5-300)	27 (3-425)	0.001
PTH (pg/mL)	33 (3.4-232)	37.5 (4-227)	35 (4-245)	NS

NS=Not significant

The median (range) level of 25-OHD was 21 (4.0-105.0) ng/mL in males and 18 (1.5-117) ng/mL in females (p=0.05). Median (range) of serum PTH in males and females was 32 (6-120) pg/mL and 36 (3.4-122) pg/mL respectively (p=NS).

Medians (ranges) of 25-OHD in spring, summer, autumn, and winter were 21 (2-300) ng/mL, 18 (3-208) ng/mL, 19 (1.5-425) ng/mL, and 17 (2-281) ng/mL respectively (p=0.04). Medians (ranges) of PTH in spring, summer, autumn, and winter were 32 (5-232) pg/mL, 32 (4-211) pg/mL, 32 (3.4-245) pg/mL, and 34 (5-220) pg/mL respectively (p=0.001). Medians (ranges) of PTH in the adult population with mild, moderate and severe vitamin D deficiencies were 28 (6-245) ng/mL, 32 (6-232) ng/mL, and 44 (3.4-222) ng/mL respectively (p=0.001).

### Prevalence of vitamin D deficiency

The prevalence of mild, moderate and severe vitamin D deficiencies among the adult population was 19.6%, 23.9%, and 26.9% respectively. However, according to the second classification, the prevalence of vitamin D deficiency (combination of moderate and severe vitamin D deficiencies or 25-OHD <20 ng/mL) and vitamin D insufficiency (mild vitamin D deficiency or 25-OHD 20-30 ng/mL) was 50.8% and 19.6% respectively.

**Fig. 1.**
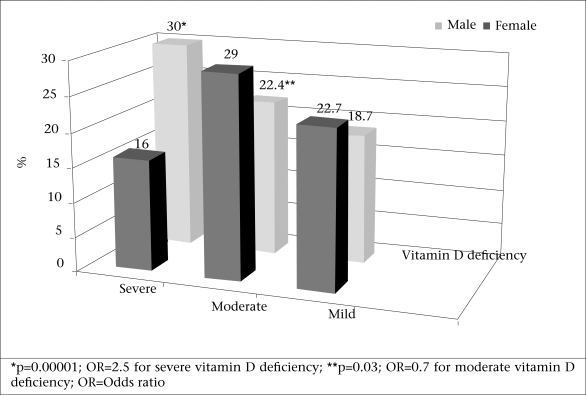
Prevalence of mild (vitamin D insufficiency), moderate and severe vitamin D deficiencies among adult males (n=243) and females (n=868)

Figure 1 shows the prevalence of vitamin D insufficiency (25-OHD 20-30 ng/mL) and deficiency (25-OHD <20 ng/mL) among males and females, according to the second classification. Therefore, the prevalence of vitamin D deficiency, according to this classification, among males and females was 45% and 52.4% respectively [p=0.04, odds ratio (OR)=0.7, χ^2^=3.9]. The prevalence of vitamin D deficiency was compared in the age-groups of 20-39, 40-59, and 60-80 years. It was higher among the younger age-group (p=0.001) (Table).

**Fig. 2.**
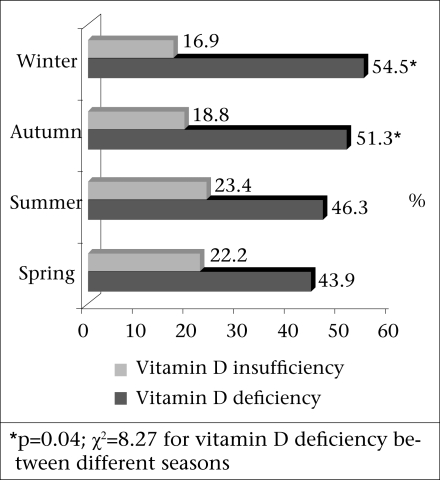
Prevalence of vitamin D insufficiency and deficiency in spring, summer, autumn, and winter

In autumn-winter, mild, moderate and severe vitamin D deficiencies were prevalent among 17.8%, 23.7%, and 30.5% of the adult population respectively. In spring-summer, mild, moderate and severe vitamin D deficiencies were prevalent among 22.7%, 24.2%, and 21.0% of the adult population respectively. The prevalence of severe vitamin D deficiency was higher in autumn-winter than in spring-summer (p=0.001, OR=1.6, 95% confidence interval 1.2-2.2). The prevalence of vitamin D deficiency was higher in colder seasons (p=0.04, χ^2^=8.27) (Fig. 2).

### Cut-off point of serum 25-OHD

**Fig. 3.**
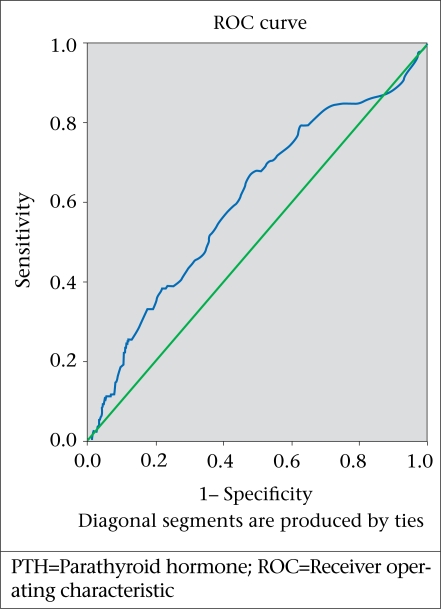
Cut-off point for vitamin D deficiency according to level of PTH

In our study population, using the receiver operating characteristic curve (ROC), the cut-off point of serum 25-OHD was determined to be 30 ng/mL (Fig. 3).

The normal range for calcium in our laboratory was 8.5-10.1 mg/dL whereas the mean (SD) of calcium in the study was 9.3 (0.62) mg/dL. A subgroup (n=66) of the 1,111 persons—9 males and 57 females—had calcium of <8.5 mg/dL. The mean age of this subgroup was 41 (median 13) years. The mean concentrations of their calcium and phosphate were 8.01 (0.56) mg/dL and 3.43 (0.89) mg/dL respectively. Medians (ranges) of their PTH and 25-OHD concentrations were 37 (5-120) pg/mL and 22 (1.5-109) ng/mL respectively.

## DISCUSSION

The results of the study in Isfahan, a sunny city located in the central part of Iran, confirms the high prevalence of vitamin D deficiency (50.8%) and insufficiency (19.6%) among the adult population and an even higher prevalence among younger adults and women. The findings showed that women were more likely to be vitamin D-deficient than men, especially for severe vitamin D deficiency (OR=1.4) (Fig. 1). However, there is no significant difference in vitamin D insufficiency between men and women. Although Isfahan is a sunny city, direct exposure to sun is, however, limited. According to legislation, all women are required to wear a scarf and long-sleeve clothes. This is why they have more severe vitamin D deficiency. On the other hand, most men wear long-sleeve shirts, especially those who work in governmental administrations. Fear from skin cancer encourages people to use anti-solar creams on their face. Living in apartments which is increasing due to increased population of the country and tendency to live in big cities are among other factors which restrict exposure to sun in Iran.

The calcium level in 66 of the 1,111 study population was of less than normal ranges, which may be due to vitamin D deficiency. The median (range) of 25-OHD in this subgroup was 22 (1.5-109) ng/mL. Thus, the cause of hypocalcaemia in some persons may not be related to vitamin D deficiency. In our study, according to the ROC, the cut-off point for 25-OHD was 30 ng/mL (Fig. 3). However, the median concentration of 25-OHD in the 66 persons was within vitamin D deficiency or at least insufficiency range.

Vitamin D deficiency is the most common medical condition worldwide. An estimated one billion people in the world have vitamin D deficiency or insufficiency (1). The prevalence of vitamin D deficiency among adult population was reported to be 14-59% with a higher prevalence in Asian countries (19-21).

Several studies in different parts of Iran and in different age-groups have shown the high prevalence of vitamin D deficiency (13-17). In a similar study in Tehran among the general population, aged 20-64 years. Hashemipour *et al.* reported that the prevalence of severe, moderate and mild vitamin D deficiencies was 9.5%, 57.6%, and 14.2% respectively (14).

Despite the fact that direct comparisons of results of different studies are difficult due to the use of different methods for the measurement of 25-OHD concentrations and that different definitions for vitamin D deficiency have been used, the findings of our study indicate that the rate of severe vitamin D deficiency status has an increasing trend.

In a study in Norway among five main immigrant groups, including Iranians, the median serum 25-OHD levels were 12.4 ng/mL and 10.8 ng/mL in Iranian men and women respectively, which was significantly lower in women than in men (22).

The prevalence of mild, moderate and severe vitamin D deficiencies was 22.3%, 25.4%, and 2% respectively in Arizona, an area with high exposure to sun, using the same definition as we had used in this study. The mean serum 25-OHD concentration was 26.1 ng/mL and lower concentration of circulating 25-OHD was observed more frequently in women (23).

In a study based on data from the National Health and Nutrition Examination Survey III (NHANES III) by Looker *et al.*, the prevalence of vitamin D deficiency (25-OHD <7 ng/mL) and insufficiency (25-OHD <25.0 ng/mL) among adolescents and adult population of the United States was reported to be 1% and 58% respectively (24).

In the present study, both median 25-OHD concentration and prevalence of vitamin D deficiency but not insufficiency were more prevalent among women than among men. Although other studies reported similar results in this field, it, however, seems that factors involved in vitamin D deficiency may be different more between men and women in some ethnic groups than others. Other factors are outdoor activity and clothing habits (veiling). Many studies have demonstrated that the rate of vitamin D deficiency in veiled women was higher (25-27).

In our study, the prevalence of vitamin D deficiency was much higher among the younger age-group whereas most studies reported the higher prevalence of vitamin D deficiency among the elderly people (28-30, 31). It may be due to supplementation of vitamin D among elderly people, especially women, who are getting used to taking multivitamin tablets. In addition to clothing habit/lifestyle, modification among younger people partly could explain the results. Younger people prefer living in apartments and have less outdoor physical activity whereas older people prefer living in houses and have had more outdoor physical activity when they were younger and also now. We did not measure height and weight of the population studied. It is one of the pitfalls of our study. If we had these parameters and calculated their BMI and its relationship with vitamin D status, it would have explained why older people have higher concentration of vitamin D. However, more studies are needed to clarify the cause.

In this study, median of vitamin D was higher in summer than in winter (Fig. 2) and the prevalence of severe vitamin D deficiency was higher in winter than in summer (OR=1.3). This finding indicates that season as an environmental factor could have effect on the severe form of vitamin D deficiency. Several studies have demonstrated that vitamin D deficiency and mean of low vitamin D level have been higher in winter. According to the study of Kull *et al*., the mean serum 25-OHD concentration in winter was significantly lower than in summer among the general population of Estonia, and likewise in our study, mean of PTH was not significantly different in two seasons (32).

It seems that factors, such as style of clothing, air pollution, skin pigmentation, and insufficient vitamin D intake, lack of routine enrichment of foods with vitamin D in Iran, could be responsible for the findings of our study (14, 15). However, not measuring the dietary vitamin D intake, duration of exposure to sunlight, and other possible risk factors for vitamin D deficiency are among the limitations of our study.

Based on the findings of the study, we recommend fortification of foods with vitamin D to treat and prevent vitamin D deficiency as the styles of clothing and other lifestyle factors are not expected to be changed in the present time.

## ACKNOWLEDGEMENTS

The authors thank Dr. Ann J. Conway for her help in English editing of the manuscript.
